# Preoperative and Operative Risk Factors for Conversion of Laparoscopic Cholecystectomy to Open Cholecystectomy in Pakistan

**DOI:** 10.7759/cureus.5446

**Published:** 2019-08-20

**Authors:** Amina Amin, Muhammad Ijlal Haider, Iram S Aamir, Muhammad Sohaib Khan, Usama Khalid Choudry, Mohammad Amir, Abdullah Sadiq

**Affiliations:** 1 General Surgery, Shifa International Hospital, Islamabad, PAK; 2 Physiology, Bahria University Medical and Dental College, Karachi, PAK; 3 Surgery, Shifa International Hospital, Islamabad, PAK; 4 General Surgery, Shifa Tameer-e-Millat University, Islamabad, PAK; 5 Surgery, Rawalpindi Medical University, Rawalpindi, PAK

**Keywords:** cholecystitis, laparoscopic cholecystectomy, ultrasonography, open cholecystectomy

## Abstract

Introduction

The currently available literature suggests a wide range of conversion (4.9-20%) from laparoscopic cholecystectomy (LC) to open cholecystectomy (OC) despite the increase in surgical expertise. Open cholecystectomy is important as the last resort for safe surgical practice in complicated cases. Increased number of pre-operative and perioperative risk factors need to be identified to pre-empt conversion. However, there has been a significant decrease in conversion rates over the past few decades. This study was conducted to determine conversion rates in our population and to identify any significant risks for conversion.

Methods

This prospective study was conducted at the Shifa International Hospital, Islamabad, Pakistan, including 1081 cholecystectomies, performed over a two-year period from January 2017 to January 2019. Comparison of risk factors between the two groups; laparoscopic cholecystectomy (LC) group and conversion to open cholecystectomy (OC) group was done. Statistical analysis was done using SPSS 24.0.1. P<0.05 were considered significant.

Results

In our study, the overall conversion rate was 7.78%. Factors of conversion to open cholecystectomy (OC) included age ≥65, morbid obesity, diabetes mellitus, and previous abdominal surgery. Deranged alkaline phosphatase (ALP), increased total bilirubin, increased common bile duct (CBD) diameter, and multiple stones in ultrasonography showed a statistically significant association with the conversion. Per-operative findings of increased adhesions >50%, empyema gallbladder (GB), perforated GB, and scleroatrophic GB showed a higher risk of conversion too (p <0.05). However, there was no statistical association with preoperative endoscopic retrograde cholangiopancreatography (ERCP) to OC in our population.

Conclusion

An open cholecystectomy is a safe approach for patients with complicated gallbladder disease. No doubt laparoscopic cholecystectomy is the gold standard having its outstanding benefits. This study identifies predictors of choice for OC in addition to the decision to convert to OC. In view of the raised morbidity and mortality associated with open cholecystectomy, distinguishing these predictors will serve to decrease the rate of OC and to address these factors preoperatively.

## Introduction

About 20 million people in the United States of America (15% of the population) have gallstones [1]. Ultrasound studies in Europe showed a prevalence of 9-21% and an incidence of 0.63/100 persons/year [2]. An annual surgical incidence of 4.2% in males and 14.2% in females was reported in a study from Pakistan [3]. Laparoscopic cholecystectomy (LC) is considered as a gold-standard treatment for cholelithiasis all around the world. Hu et al. reported the conversion rate from laparoscopic cholecystectomy to open cholecystectomy (OC) to be 1-15% [4]. Tayeb et al. from Pakistan reported the conversion rate to be 7.2% a decade ago [5]. Currently, there has been no analysis from our part of the world stating the preoperative and operative risk factors involved in the conversion of laparoscopic cholecystectomy to open cholecystectomy. A greater understanding of the pre-operative and intra-operative factors, leading to conversion, are important to be known. Understanding outcomes is essential for advancing health care, and conversion to open cholecystectomy will always be necessary for safe surgical practice [6]. Plausible reasons for conversion include inflammation and dense fibrosis of the Calot’s triangle, ambiguous anatomy, life-threatening bleeding, and bile duct injury [7]. As expected, apart from the loss of all the potential benefits of a minimally invasive procedure, the conversion also causes a prolonged hospital stay, increased morbidity, and an increased cost. Based on the risk factors evaluated from our study, a prediction model can prevent the surgeons from persisting with a difficult operation.

## Materials and methods

This prospective observational study was conducted at the Shifa International Hospital, Islamabad, Pakistan, involving all patients presenting with symptomatic gallstones in the emergency or outpatient department. The study was conducted over a period of two years from January 2017 to January 2019. Written and informed consent was taken. Patients with other known concomitant infective etiology, immunocompromised state, and any proven malignancy were excluded from the study. All the patients were planned for laparoscopic cholecystectomy. The data of preoperative and perioperative factors were collected on a standard proforma. Preoperative factors included age, morbid obesity, uncontrolled diabetes mellitus, previous upper abdominal surgery involving any surgical incision in upper six abdominal quadrants, previous endoscopic retrograde cholangiopancreatography (ERCP), total leukocyte count (TLC), liver function tests (LFTs) including aspartate transaminase (AST), alanine transaminase (ALT), alkaline phosphatase (ALP), total bilirubin, and severe sepsis. Ultrasonographic findings such as common bile duct (CBD) diameter, wall thickness, and the number of stones were recorded. Operative factors included gallbladder (GB) adhesions and gross appearance of the gallbladder (scleroatrophic, empyema, perforated). The dependent outcome variable was conversion to open cholecystectomy. For analysis, the patients were divided into two groups according to the age; taken as >65 and <65 years. Morbid obesity was also classified into two based on the presence or absence of morbid obesity defined as a body mass index (BMI) of >39.9 kg/m^2^. Presence of uncontrolled diabetes mellitus was taken as a medical risk factor in co-morbidities. Laboratory values of liver function tests and total leukocyte count were categorically recorded preoperatively as per abnormal for our institution laboratory. Radiology report of preoperative ultrasonography was analyzed for three separate factors (CBD diameter, GB Wall thickness >4mm, and the number of gallstones). Preoperative ERCP was also included as one of the risk factors for patients who were diagnosed with choledocholithiasis and underwent ERCP. Operative findings, as noted by the operating surgeon, were compared to identify the factors related to conversion. The extent of adhesions on the gallbladder was categorized into less than and more than 50%. Gross appearance of the GB was taken as another risk factor which included scleroatrophic GB (scarred and contracted GB), empyema (pus in GB), perforated GB (contents of GB if seen in the peritoneal cavity). Statistical analysis was done using the Statistical Package for Social Sciences (SPSS) v.24.0.1. For comparison between the two groups, the Chi-square test was used. Odds ratio (OR) and confidence interval (CI) were calculated for all the variables. A p-value <0.05 was considered statistically significant.

## Results

A total of 1081 patients were included in the study. All patients underwent laparoscopic cholecystectomy. A total of 997 (92.22%) patients underwent laparoscopic cholecystectomy successfully, while 84 patients had to undergo conversion to open cholecystectomy. The laparoscopic to open conversion rate was 7.78% in our population. The mean age of patients was 41±15.6 years. The mean BMI was 28.38±7.67 kg/m^2^. Several factors were considered and compared in the study population. Patients in the higher age group, more than 65 years, were at high risk for conversion to open cholecystectomy (OR 7.28, CI 4.25-11.59, p <0.01). Patients with morbid obesity were 5.7 times more at risk of conversion to open cholecystectomy (p <0.01). There was also a high risk of conversion to OC in diabetes (OR 2.19, CI 1.38-3.40, p <0.05). A previous upper abdominal surgery was also statistically significant with OC (OR 4.82, CI 3.04-7.65, p <0.01). There was no causal association of an elevated AST or ALT with OC (p = 0.87). However, elevated ALP (OR 5.36) and increased total bilirubin, >3mg/dl, were associated with a higher risk of open cholecystectomy (p-value <0.01). Wall thickness >4mm (OR 1.98), adhesions more than 50% (OR 5.5), and gallbladder empyema (OR 1.78) were significantly associated with a higher risk of open cholecystectomy (p <0.05). There was no statistical association of preoperative ERCP, adhesions less than 50%, and septicemia with the conversion to open cholecystectomy (p >0.05). Regarding ultrasonographic findings of multiple gallstones (OR 1.8, CI 1.02-3.16, p = 0.0395) and CBD diameter of more than 6mm (OR 6.47, CI 4.03-10.36, p <0.01), there was a higher risk of conversion. Additionally, the risk of open cholecystectomy was higher in the scleroatrophic gallbladder (OR 3.39, CI 1.93-5.94, p <0.01) as well as perforated gallbladder (OR 3.71, CI 1.76-7.79, p <0.05). The results are shown in Table 1.

**Table 1:** Comparison of various risk factors for conversion of laparoscopic cholecystectomy to open cholecystectomy ALT: Alanine transaminase; ALP: Alkaline phosphatase; TLC: Total leukocyte count; ERCP: Endoscopic retrograde cholangiopancreatography

Risk factors	Laparoscopic cholecystectomy group	Conversion to open cholecystectomy group	Risk Estimate	Confidence Interval	p-value
Age more than 65	14.74% (n = 48)	55.95% (n = 47)	7.28	4.57 - 11.59	<0.01
Morbid obesity	20.46% (n = 204)	59.92% (n = 50)	5.71	3.60 - 9.57	<0.01
Diabetes mellitus	42.52% (n = 424)	61.90% (n = 52)	2.19	1.38 - 3.40	<0.05
Previous abdominal surgery	15.04% (n = 158)	47.31% (n = 40)	4.82	3.04 - 7.65	<0.01
ALT	28.18% (n = 281)	27.38% (n = 23)	0.96	0.58 - 1.58	0.87
ALP	22.36% (n = 223)	60.71% (n = 51)	5.36	3.37 - 8.51	<0.01
Total bilirubin	24.57% (n = 245)	55.95% (n = 47)	3.89	2.74 - 6.14	<0.01
TLC	33.80% (n = 337)	51.19% (n = 43)	2.05	1.31 - 3.20	<0.05
Wall thickness	37.91% (n = 378)	52.87% (n = 46)	1.98	1.20 - 3.10	0.0028
Multiple stones	70.21% (n = 700)	80.95% (n = 68)	1.80	1.02 - 3.16	0.0395
Pre-ERCP	42.22% (n = 421)	47.61% (n = 40)	1.24	0.79 - 1.94	0.338
Adhesion<50%	67.70% (n = 675)	17.85% (n = 15)	0.103	0.05 - 0.18	<0.01
Adhesion>50%	20.86% (n = 208)	57.14% (n = 48)	5.5	3.19 - 7.99	<0.01
Empyema	16.54% (n = 165)	26.19% (n = 22)	1.78	1.06 - 2.99	<0.026
Scleroatrophic gallbladder	5.51% (n = 55)	35.71% (n = 30)	9.51	5.64 - 16.04	<0.01
Common bile duct diameter	21.76% (n = 217)	64.28% (n = 54)	6.47	4.03 - 10.36	<0.01
Perforated gallbladder	3.51% (n = 35)	11.90% (n = 10)	3.71	1.76 - 7.79	<0.05

## Discussion

Advantages of the laparoscopic cholecystectomy make it a gold standard for symptomatic gallstones till date. It is a safe and cost-efficient procedure [8]. Identifying the reliable risk factors will benefit patient education and post-operative outcome. A total of 1081 patients were enrolled in the study over a period of two years. In our study, the conversion rate was 7.78%. In a study by Rosen et al., the conversion rate was reported to be 5.3% [8]. Advancing age, more than 65 years, was associated with a high conversion rate (OR 7.28). Sippey et al. also reported increasing age as a factor for conversion (OR 1.01) [9]. This can be explained by the difficult dissection secondary to a repeated number of attacks of inflammation and ensuing thickening and fibrosis. In our study, morbid obesity has proven to be a significant factor for conversion to open cholecystectomy (OR 5.71). Goonawardena et al. also reported BMI >30 as a risk factor for conversion [10]. There can be several underlying factors obliterating the proper anatomy, such as short trocar length, port displacement, and increased fat deposition at the Calot’s triangle.

Another risk factor for conversion to open cholecystectomy was a history of previous upper abdominal surgery (OC 47.31% vs. LC group 15.72%). Akyurek et al. also reported a significant conversion rate with the history of prior upper abdominal surgery [11]. This increased conversion rate was due to inaccessibility of Calot’s triangle secondary to adhesions with the surrounding structures. Uncontrolled diabetes also has an association as a risk factor for conversion (p-value <0.05) [12-14]. However, the exact underlying hypothesis for this finding is not yet known. Ultrasonographic signs of inflammation such as wall thickness, multiple GB stones, and increased CBD diameter are risk factors for conversion. Thickened GB walls indicate chronic inflammation and repeated attacks of cholecystitis, which were associated with conversion. A few other studies supported this finding [14-17].

Sultan et al. reported a conversion rate in patients with multiple gallstones, on ultrasound finding, to be 83.3% compared to our study, which was 80.95% [17]. Increase CBD diameter on ultrasound is a sign of choledocholithiasis. A study reported that choledocholithiasis is associated with a higher risk of conversion (OR 6.9), which was comparable to our study (OR 6.47) [15]. In our study, hematological investigations like TLC and liver function tests showed a significant association with conversion to open. Biochemical markers of obstructed GB such as ALP and raised total bilirubin proved to be an important risk factor in conversion [12-14]. A study reported conversion rate, increasing up to 20.2% in patients with deranged ALP and leukocyte count [18]. However, AST and ALT didn’t show any association with conversion to OC, neither there was any study to support this, to the best of our knowledge. Gross appearance of the gallbladder does impart some knowledge based on the experience of the surgeon that how easy or difficult the cholecystectomy may be. Surgery on GB with multiple gallstones and a scleroatrophic GB is a challenge due to numerous factors. The gallbladder is hard to grip due to fibrotic tissue, resulting from chronicity secondary to cholelithiasis, intrahepatic gallbladder, or short cystic duct [19]. So, the two variables of gross appearance in our study, high-grade adhesion and scleroatrophic gallbladder were found to be associated with conversion in our population. Ercan et al. documented that an increased number of adhesions was related to a higher chance of conversion (OR 4.76), which was comparable to our data (OR 5.50) [7]. Preoperative ERCP is indicated when there is prior evidence of choledocholithiasis. The laparoscopic cholecystectomy was undertaken in such patients till 72 hours of the post-procedure due to the formation of adhesions as a sequela of dense inflammation. Ercan et al. also reported preoperative ERCP as one of the factors for conversion to open (OR 1.83), which was relatable in our data (OR 1.24) [7].

Our study limitations included observational study design pattern and limited sample size of open cholecystectomies. We recommend a study of a larger cohort of open cholecystectomies with better study design.

## Conclusions

The conversion rate from LC to OC was very low, at 7.78%, in our population. It lies within the lower range of previously reported conversion rates. This is likely multifactorial and reflects an increase in the expertise of the laparoscopic technique, better quality of laparoscopic instruments all around the world, and an enhanced understanding of the existing knowledge of risk factors involved in the conversion. An open cholecystectomy is a safe approach for patients with complicated gallbladder disease. With its outstanding benefits, laparoscopic cholecystectomy is undoubtedly the gold standard. This study identifies predictors of the choice for OC in addition to the decision to convert to OC. In view of the raised morbidity and mortality associated with open cholecystectomy, distinguishing these predictors will serve to decrease the rate of OC and to address these factors preoperatively.
